# Applications of a formal approach to decipher discrete genetic networks

**DOI:** 10.1186/1471-2105-11-385

**Published:** 2010-07-20

**Authors:** Fabien Corblin, Eric Fanchon, Laurent Trilling

**Affiliations:** 1 Laboratoire TIMC-IMAG, UMR CNRS/UJF 5525, Domaine de la Merci, 38710 La Tronche, France; 2 Laboratoire IRISA-INRIA centre de Rennes, Campus de Beaulieu, 35042 Rennes, France

## Abstract

**Background:**

A growing demand for tools to assist the building and analysis of biological networks exists in systems biology. We argue that the use of a formal approach is relevant and applicable to address questions raised by biologists about such networks. The behaviour of these systems being complex, it is essential to exploit efficiently every bit of experimental information. In our approach, both the evolution rules and the partial knowledge about the structure and the behaviour of the network are formalized using a common constraint-based language.

**Results:**

In this article our formal and declarative approach is applied to three biological applications. The software environment that we developed allows to specifically address each application through a new class of biologically relevant queries. We show that we can describe easily and in a formal manner the partial knowledge about a genetic network. Moreover we show that this environment, based on a constraint algorithmic approach, offers a wide variety of functionalities, going beyond simple simulations, such as proof of consistency, model revision, prediction of properties, search for minimal models relatively to specified criteria.

**Conclusions:**

The formal approach proposed here deeply changes the way to proceed in the exploration of genetic and biochemical networks, first by avoiding the usual trial-and-error procedure, and second by placing the emphasis on sets of solutions, rather than a single solution arbitrarily chosen among many others. Last, the constraint approach promotes an integration of model and experimental data in a single framework.

## Background

A central task in molecular systems biology is to build and analyze genetic and biochemical networks in order to decipher the properties of cellular phenomena. The emphasis is not on investigating in detail one or a few molecules at a time, as is done traditionally in molecular biology, but rather on focusing on the network level.

We are specifically interested here in gene regulatory networks (GRNs) formalized as discrete genetic networks as defined by R. Thomas [1,2]. The main goal of this formalism is to obtain a qualitative understanding of the network dynamics by reasoning on discrete entities. In GRNs the molecular players are the genes and the proteins they produce. A genetic interaction corresponds to the fact that a gene *g_i _*produces a protein *p_i _*which influences the expression rate of another gene *g*_*j*_, or *g_i _*itself. The set of all these genetic interactions constitute the interaction graph, to be defined formally later. In a given state of the network, each gene has a certain expression rate (the rate of production of the encoded protein) which depends on the presence or absence of a subset of proteins, the activators or repressors of the considered gene. The expression rate of a gene is maximum when all its activators are present and all its repressors are absent. In this context a basic objective is to analyze the temporal evolution of the protein concentrations in given external conditions. This can be done when the values of the model parameters have been measured. When this is not the case, the problem is then to exploit the knowledge about network behaviour (e.g. response to perturbations, phenotype change when one or several genes are knocked-out) to deduce the possible values of the parameters. A frequently used method consists in performing a large number of simulations by varying some parameters (generally one or two at a time) and selecting a posteriori the set of values that is consistent with the observed behaviour. As explained below, we propose a different approach for this inference problem.

Beyond these basic functionalities (simulation, inference of parameter values), the construction of GRN models consistent with experimental data requires more sophisticated tools. It often occurs that a proposed model displays inconsistencies with part of the data. In such cases it is necessary to critically analyze the hypotheses used in building the model and to revise them. This analysis can be done "by hand" for small networks, e.g. up to three genes, but requires the use of computational tools to cope with the complexity of larger networks. In still other situations the observational constraints are weak with respect to the number of variables, and the number of solutions is very large. In such cases, it is interesting to derive properties that are shared by all the solutions, or subsets of them, in order to get a better understanding of the model properties and to design new experiments having the potential to substantially reduce the set of solutions.

Fundamentally, we want to provide the biologist studying GRNs with a software environment allowing to perform such tasks. The available knowledge is partial and bears on both the *structure *of the network of interest (the set of interactions) and the *behaviour *of the network in various conditions. The first kind of knowledge is said to be structural, or local (each interaction is a piece of information and can be studied in itself), whereas the second kind is said to be behavioural, or global (the network as a whole is giving rise to a given behaviour). The network architecture and its behaviour are closely inter-related. This relation is implemented formally as a set of constraints in a straightforward manner in our software environment, named GNBox (Genetic Networks toolBox - Additional file 1). More precisely, the philosophy of this approach is to represent a given problem, or set of problems, as a set of formulae linking variables. In our case this entails the specification of (i) the rules defining the updating scheme (how the successors of a state are computed); (ii) the network architecture (set of interactions); (iii) the observations about the behaviours of the network (partial information about paths), or any working hypothese about the system; (iv) the query itself (e.g. number of stationnary states, possible values of initially unkown parameters). The set of constraints thus defined is then submitted to a solver whether there exists solutions or not. A distinctive feature of the constraint approach is constraint propagation. It implements deduction rules and allows in favorable conditions to reduce drastically the search space, thus limiting enumeration. Of course some amount of enumeration is usually still necessary, but the aim of the game is to reduce it as much as possible. This formal relation is "executable" and allows not only to perform basic functionalities such as simulation or reverse-engineering, but also to assert and obtain properties on both the behaviours and the interactions. More specifically, we implemented in this constraint-based setting four main functionalities: (i) proof of consistency or inconsistency of a constraint pool, (ii) constraint relaxation in case of inconsistency (model revision), (iii) prediction of properties in case of consistency, (iv) search for minimal models, with respect to the number of thresholds, for example.

In this article we present our approach and we show how it can be applied successfully to the analysis of three different biological problems. In the section Methods we present the formalism we developped. We present the formal definition of interaction graphs and of the evolution rules of Thomas networks. These notions are required to express the queries implementing the functionalities mentionned above. Other notions related the specification of interaction compositions facilitate the expression of properties involving kinetic parameters. The implementation is discussed in [3]. In the section Results and Discussion we present three applications which differ by both the type of knowledge available and the type of biological questions asked. These applications permit (i) to illustrate the different functionalities of GNBox, (ii) to show the feasibility of this constraint-based approach on realistic biological problems, and (iii) to support the idea that a formal and declarative approach is very interesting to decipher the properties of GRNs, in order to assist in their construction.

## Methods

We present briefly in this section the constraint technology, the constraint-based formalization of Thomas networks, the constraint-based formalization of biological properties of these networks, and the features of our software environment GNBox to elucidate GRNs.

Below we use the following mathematical notations: an integer *x *taking values between *min *and *max *is denoted *x *∈ *min..max*, a Boolean *b *is an integer such as *b *∈ 0..1, *b*1 ⇔ *b*2 means that the Boolean *b*1 is equal (or equivalent) to the Boolean *b*2, *b*1 implies *b*2 is denoted *b*1 ⇒ *b*2, the Boolean equal to *b*1 and *b*2 is denoted *b*1 ∧ *b*2, the Boolean equal to *b*1 or *b*2 is denoted *b*1 ∨ *b*2, the Boolean equal to the conjunction of a list of Boolean *b_i _*is denoted ∧ *_i _b_i_*, the Boolean equal to the disjunction of a list of Boolean *b_i _*is denoted ∨_*i *_*b*_*i*_.

### Constraint technology

We propose to implement the approach (particularly the link between network structure and behaviour) using Constraint Logic Programming (CLP) technology, with a finite domain solver. CLP is a programming paradigm based on first order logic. CLP considers specific classes of logical terms and proposes efficient resolution methods of equations over these terms (constraints). A CLP program is a logic formula, and its execution is the construction of a proof of consistency (or inconsistency) of this formula. The formula is consistent when it is possible to find an instantiation of the variables which satisfies the formula. Logicians call such an instantiation a model. A CLP program is reversible in the sense that it permits to impose and obtain partial knowledge over all the variables of the formula (including in our case the variables describing the interactions and behaviours). For example, let say that *p*(*x*, *y*) is a predicate defining a relationship between two entities *x *and *y*. If a measurement allows to reduce the domain of values of *x*, this information can be added as an additional constraint, and a *query *can be submitted about the possible values of *y*. The solver will try to propagate the additional information on *x *to reduce the domain of *y*, taking into account *p*(*x*, *y*). Conversely, if the measurement has been performed on *y*, this information can be propagated to *x *through *p*(*x*, *y*). This is reversibility. It must be said that different kinds of solvers exist, characterized by the type of variables (e.g. finite domain integers, reals) and the type of propagation rules used, among other things.

As all the variables describing interactions and behaviours have finite integer domains in the discrete framework used, the use of a constraint solver over finite domains is very well suited. In addition, the expressive power of first order logic and constraints over integers allows the definition of very general properties and functionalities. Finally, in order to be able to take advantage of the very efficient Boolean Satisfiability (SAT) solvers available, the GNBox environment is able to translate the CLP formalization into a Boolean formula in Conjunctive Normal Form (conjunction of disjunctions of Boolean variables or their negation, the input format used by most SAT solvers). Details on the translation into CNF can be found in [3]. In this way we combine the expressive power of CLP with the efficiency of SAT solvers.

### Formalization of Thomas networks

**Figure 1:**
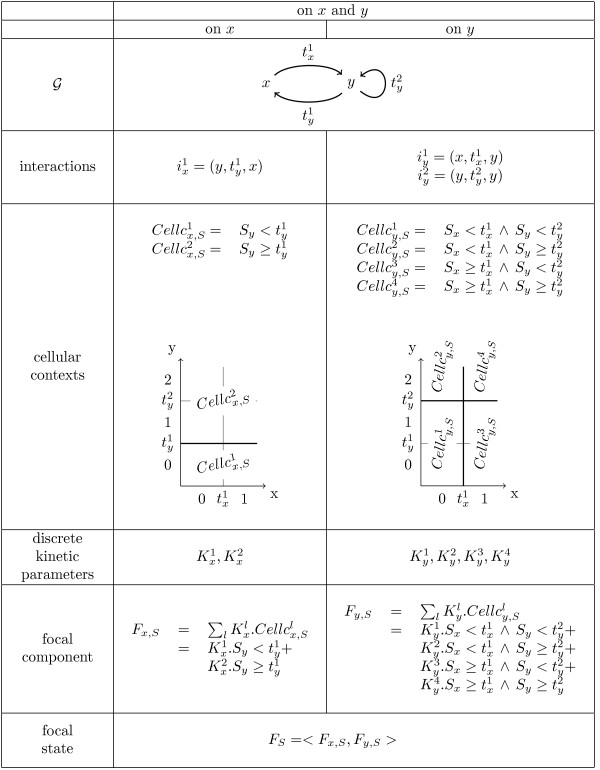
**Illustration of main notions defining a model *M *from an example of interaction graph **.

**Figure 2:**
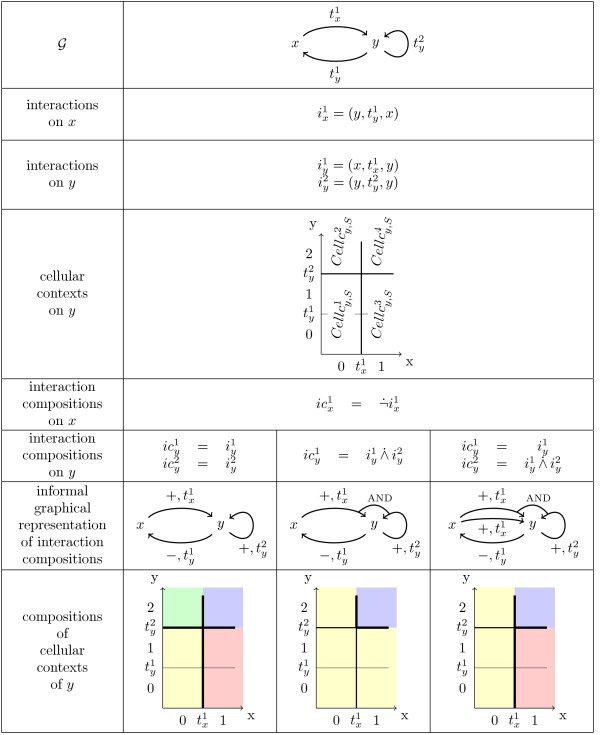
**Examples of interaction compositions and resulting compositions of cellular contexts over example in Figure 1**.

In this subsection we present a constraint-based formalism to impose, check and infer properties about discrete genetic networks as defined by R. Thomas. We first introduce the notions needed to define and formalize the interaction graph and the evolution rules of Thomas networks. We define in the next subsection the notions of composition of interactions, additivity and observability properties which are useful to express hypotheses about kinetic parameters. All the presented notions of this subsection and the next one are illustrated with the example of Figure 1 and Figure 2, and will be put into use in the biological applications of the section Results.

The structure of a GRN is represented in an abstract way by an interaction graph . The nodes of  are genes. Each node is associated to a concentration variable representing the concentration of the protein produced by the corresponding gene. The oriented edges of  represent *interactions *between these genes, denoted by  for the interaction on the gene (component) *c *of index *r*, *r *∈ 1..*r_c_*, where *r_c _*is the number of interactions on *c*. An integer variable representing a discrete *threshold *labels each edge. In papers using the formalism of R. Thomas edges in interaction graphs are also labeled by a sign [1]. We choose more primitive interaction graphs without these labels in order to generalize the formalization and to facilitate the expression of hypotheses about the way interactions compose on target nodes. The presence of an interaction from gene *a *to gene *b *(with a threshold *t*) indicates that protein *a *can potentially modify the expression rate of gene *b*. Furthermore, this change in expression rate, when it actually exists, takes place when concentration of *a *crosses threshold *t*. In other words, this interaction indicates that the rate of production of protein *b *can be influenced by the position of protein *a *with respect to threshold *t*. It has to be noted that such an interaction does not actually impose a difference in production rate. Rather, the absence of such an interaction forbids the existence of such a difference in production rate. Such an interaction is represented by the triplet (*a, t, b*) (labelled edge). In the example in Figure 1, the rows "" and "interactions" give respectively the definition of the interaction graph  and the sets of interactions for the target genes *x *and *y*.

The network structure being defined, the next step is to define the network state and dynamics. A *state S *of the network is a list of gene product concentrations (protein or RNA). The concentrations are discretized according to the thresholds appearing in . The concentration of the product of gene *c *in state *S *is the integer *S_c _*∈ 0..*max_c_*, where *max_c _*is the maximal value of the discrete concentration of protein *c*. The threshold of component *c *of index *p *is  ∈ 1..*max_c_*, where the index *p *takes its values in 1..*max_c _*(obviously if a concentration is cut by *max_c _*thresholds the associated discretized variable will take *max_c _*+ 1 values). For a given system with *n *genes, *c_i_*, *i *∈ 1..*n*, is the variable associated to the *i^th ^*gene, and state *S *is the ordered list . So, the discrete concentration space contains  states.

We are now in a position to explain how the successor states of a given state are computed. Each state *S *is associated to a so-called *focal state*, denoted by , and belonging to the same state space. The focal state gives the direction of evolution (tendency) for each concentration. Consider for instance *S *= ⟨0, 0⟩ and *F_s _*= ⟨1, 1⟩ in a 2-dimensional system. The successor of *S *is not ⟨1, 1⟩ as is the case in synchronous updating schemes. Rather *F_S _*= ⟨1, 1⟩ indicates the direction of evolution of each component taken separately. Here both are increasing and *S *= ⟨0, 0⟩ has 2 successors, ⟨1, 0⟩ and ⟨0, 1⟩. In other words two transitions are possible from *S*, and this type of updating scheme is often called asynchronous, but nondeterministic is a better term. What is the basis of this non-determinism? If the numerical values (real numbers) of the initial concentrations, together with those of the model parameters were known, it would be possible to determine the exact successor. In the discrete abstraction considered here this information is not available and consequently both possibilities must be taken into account. Non-determinism is a fundamental property of this abstraction due to the information loss induced by the partition of concentration space into rectangular domains. We chose this formalism in this study because it is well founded and it is a good match to the qualitative knowledge generally available in Systems Biology at present. Nevertheless, it should be kept in mind that our constraint approach is not tied to Thomas networks. Other types of discrete dynamical rules could be implemented, e.g. Kauffman-like Boolean networks with parallel (synchronous) [4] or block-sequential updating scheme [5,6].

To implement the Thomas evolution rules we need first to specify the equations which link a state *S *to its focal state *F_S_*. These equations are named *focal equations*. A set of rules then links state *S*, the focal state *F_S _*associated to *S*, and the successor states of *S*. We stress here that these rules must be viewed as relationships linking different kinds of unknowns. As explained above (reversibility), the use of these relationships depends on the available information in a given state of knowledge. If the concentration values making state *S *are all known, together with the position of its focal state, then the successors of *S *can be computed. But the relationships can be exploited in other ways, too.

The system of focal equations contains different kinds of parameters: constant concentrations associated to *input genes *(that is genes that are influenced by no genes in the network and whose state is fixed by external conditions), parameters related to reaction kinetics (similar to those that would appear in a differential description), and thresholds . The set of all these parameters is denoted by *P*. The parameters are amongs the unknowns of the system of constraints because their values are in general not known, or only partially known. The evolution rules, once formalized (see below), lend to a first set of logical constraints. To this first set are added structural constraints over the parameters derived from experimental data, and working hypotheses. The set of solutions of such a system of constraints defines a set of instantiated models (i.e. models in which all parameters are instantiated). The couple composed of a focal equation system and a set of structural constraints is called a *parameterized constrained model M*, or just *model*. A typical query includes one or several structurally-related models (when data are available on several mutants), and some additional behavioural constraints. If the resulting system (set of all constraints of the query) is under-constrained this set contains a large number of solutions. If it is over-constrained it is empty. In our context, this last case is interpreted as a contradiction between, on one hand, the experimental evidence and, on the other hand, the network structure or the hypotheses. More sophisticated queries are presented in the application part below, to illustrate the high-level functionalities mentioned in the introduction.

The parameterized focal equation system of a model *M *is completely defined by an interaction graph . In fact these two entities contain exactly the same information (as long as kinetic parameters are not instantiated nor constrained). The set of interactions of  having the gene *c *as target induces a partition of the concentration space according to the thresholds  of these interactions. This partition defines a set of regions called the *cellular contexts *of *c*. As long as the concentration of the proteins *c' *regulating *c *do not cross one of the  thresholds, the system stays in the same cellular context, because from the viewpoint of gene *c *the regulatory conditions have not changed. This means that all the states *S *belonging to the same cellular context of *c *have the same focal component *F_c,S _*of the focal state *F_S_*. The value of *F_c,S _*being generally unknown, a formal parameter  called *discrete kinetic parameter *is introduced for each cellular context of *c *with index *l*. These parameters are the discrete version of the ratio of protein production rate over degradation rate. When the value of  is high in some cellular context, this is interpreted by saying that in the states belonging to that context the production rate of the protein associated to gene *c *is high, and/or its degradation rate low. But in the qualitative setting of Thomas formalism it should be kept in mind that we have only access to a discretized version of the production rate to degradation rate ratio. The number of cellular contexts for a given gene *c *is , and so is the number of  parameters.

We have introduced the main notions unformally (interaction, threshold, state, focal state, focal equation, cellular context, discrete kinetic parameter), and will now present formal definitions which are directly usable in constraint form.

**Definition 1 ***Let c be a component, and let S be a state. The **focal component **of c in S, denoted by F_c,S_, is defined by the following **focal equation **of c:*.

*where ** is the **discrete kinetic parameter **of c with index l, l ∈ *1*..l_c_, and  is a condition true if S belongs to the **cellular context **of c with index l. The indexing convention is the following: l is equal to V *+ 1 *where V is the decimal value of the binary number composed of the Booleans  with , these Booleans being arranged in increasing order of r (this is just meant at providing a unique numbering of the cellular context and is not fundamental)*.

The above formula means that if state *S' *belongs to the cellular context of index *l' *for gene *c *(that is  is true) then the focal component *F_c,S' _*is equal to .

**Example 1 ***The row "cellular contexts" in *Figure 1* describes formally and graphically, for a given order of thresholds, the cellular contexts for each component x and y of the considered example. Component x is the target of only one interaction and is thus associated to two cellular contexts, y is the target of two interactions and has 4 cellular contexts. The row "discrete kinetic parameters" gives the list of these parameters. The subscripts and superscripts make the correspondence with the associated cellular contexts (** with **, etc.). Finally the row "focal component" gives the equations describing the focal components F_*x*, *S *_and F*_*y*, *S *_*of a state S. The row "focal state" in *Figure 1* describes the focal state F_S _*= ⟨*F_x,S_*, *F_y,S_*⟩ *of S*.

The focal state defines the direction of the dynamic transitions starting in *S*. In the Thomas networks, the authorized transitions are such that:.

1. *S' *and *S *are the same state or are neighbors,

2. *S' *and *S *differ on at most one component.

3. *S' *is in the "direction" of the focal state *F_S_*.

The first property (formally ) is due to the fact that the concentrations evolve continuously, thus jumps over states are not allowed. The second is commonly called asynchronicity. The third one is specific to the discretization of evolution equations due to Thomas. We explained above that when two concentrations are increasing in a given state, it is not known in this kind of abstraction which will reach first its next threshold, and consequently which transition will occur first. In this situation both transitions are taken into account leading to two successors for the state considered (of course this generalizes to more than two). This is intimately connected to the non-determinism inherent to abstractions based on phase space partition.

The rules have the following consequences: *S *= *S' *⇔ *F_S _*= *S *(stationarity of *S*),  = *S_c _*+ 1 ⇒ *F_c,S _*>*S_c _*(rising transition according to *c*) and  = *S_c _*- 1 ⇒ *F_c,S _*<*S_c _*(downward transition according to *c*).

It is possible to specify a knock-out or ectopic expression *mutation*. For each non-input mutated gene *c *set to a constant value *v*, the constraint ∧_*l *_= *v *must be introduced. For a mutated input gene to the value *v *the input parameter of the model is set to this value *v*. In some cases it is necessary to use several models in the same query, one model corresponding to the wild type and the others to mutants. In such cases we introduce constraints specifying that for all couples of models (*M^α^*, *M^β^*) the thresholds of *M^α ^*are equal to those of *M^β^*, and the parameters  of *M^α ^*associated to genes *c *which are not mutated in *M^α ^*and *M^β ^*are equal to those of *M^β^*. The constraints between the input parameters of *M^α ^*and *M^β ^*depends of the considered biological application (see Constraint 4, in the section Results and Discussion).

A user of GNBox must describe the structure of the studied GRN (possible interactions between genes), and can use the language *LG*1 to specify the existence of a behaviour. The language *LG*1 is composed of the predicate *path*(*M*, *Path*, *L*) which is true if *Path *is a succession of *L *states authorized by the model *M *(achieving a formal link between a model and its behaviours), and a language to impose arithmetic constraints between variables of *Path*. Language *LG*1 is used to formalize observations on the behaviour of the system. Our approach allows to specify (declare) partial information. For example only a few concentrations may have been measured. Absence of information is absence of constraints.

### Interaction compositions

The interaction graph  lists the interactions individually but does not contain information on the manner in which different interactions are composed when they have the same target gene. The information about the way to compose interactions is embodied in relationships linking the parameters contained in . However, the manual interpretation of instantiations or properties over parameters of *P *is not convenient, especially for users not acquainted with the formalism of Thomas networks. For this reason we designed a higher level language *LG*2 to impose, check and infer properties about the way to compose the interactions in  in the manner of the traditional notion of the logic of regulation (NEG, AND, OR gates). It should be understood that this is not fundamental to the approach but merely a facility to handle relationships between parameters induced by the composition of interactions. The user always has the choice to work directly on these relationships.

We explained above that the specification of a set of interactions for a gene *c *partitions (in cellular contexts) the concentration space by hyperplanes (corresponding to thresholds of interactions acting on *c*). *LG*2 permits, for every *c*, to partition the concentration space in union of cellular contexts of *c*, named *compositions of cellular contexts*, via the definition of *interaction compositions*. Any union of cellular contexts can be specified, and in particular an union of disconnected regions. Similarly to the semantic of a set of interactions, the semantic of a set of interaction compositions is the following: all the states belonging to a given composition of cellular contexts of *c *have the same evolution tendency of the concentration of protein *c*. The borders between these regions are constituted of parts of threshold hyperplanes of interactions taking part in the composition. We name these borders interaction compositions. An interaction composition for *c*, denoted by , *rc *∈ 1..*rc_c_*, *rc_c _*being the number of interaction composition on *c*, permits to indicate where it is possible to have a change in the evolution trend of component *c*. Informally, one can see an interaction composition as a new artificial species which interacts on *c *and which induces a new partition of state space into two regions. First, let us remark again that an interaction  induces a partition of state space into two regions by the hyperplane associated to the threshold . By convention, the part where the states *S *are such that  is true is said to *satisfy *. An interaction composition also partition the state space into two regions, but the border is not necessarily a hyper-plane defined by a single threshold. An interaction composition can have the following forms:

• an interaction .

• . The region where the state *S *are such that  is said to satisfy .

• , where *ic *and *ic' *are interaction compositions. The region where the states *S *satisfies both *ic *and *ic' *is said to satisfy .

• , where *ic *and *ic' *are interaction compositions. The region where the states *S *satisfies *ic*, or *ic'*, or both, is said to satisfy .

**Example 2 ***The sixth row in *Figure 2* gives three possible sets of interactions compositions for y, related to the example in *Figure 1*. The four first rows recall the context of example in *Figure 1*. The fifth row gives the set of interaction compositions over x. The seventh row shows for each of these couple of sets a graphical representation of the detailed structure of the network, with signs *+ *and - over interactions and bridges, denoted by AND, to express a conjunction between two interaction compositions. Finally, the last row shows the resulting compositions of cellular contexts for y*.

*The first case (second column of last row) leads to the same partition of the discrete concentration space of y (the areas described by the cellular contexts are the same that those described by the compositions of cellular contexts)*.

*The second case expresses with the sole interaction composition on y, , that either the concentration of x and y are above  and , respectively (the tendency of y is unique in this region), or the concentration of x or y are below  and , respectively (the tendency of y is unique in this region)*.

*The third case expresses quite the same of the second case but permits that x interacts on y whatever the concentration of y. So, we obtain three compositions of cellular contexts because the fact that x can interact on y all along the border of the threshold *.

**Figure 3:**
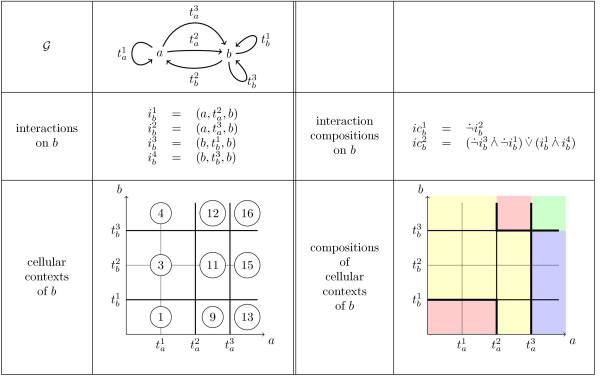
**Example of interaction compositions and resulting compositions of cellular contexts for a given order of thresholds**.

**Example 3 ***The *Figure 3* gives an example of a set of interaction compositions and resulting composition of cellular contexts. In the first column, we can see an interaction graph  with two components a and b, a set of four interactions over b, and a partition of the concentration space into nine non empty cellular contexts. The indexes l of the conditions Cell appear in circles. The other cellular contexts are empty according to the order of the values of the thresholds . Note that usually this order is not known and the values of thresholds for a same species can be equal. In the second column (to make a parallel with the interactions and cellular contexts) we assumed to have two interaction compositions. We obtain a partition of the concentration space into four non empty compositions of cellular contexts (the pink region being the union of the two disconnected cellular contexts *1 *and *12*)*.

### Additivity and observability properties

The language *LG*2 allows to define specific effects of an interaction composition on a component *c*. Here by effect we mean a shift in the position of the focal component *F_c _*when the border associated to the interaction composition is crossed. Biologically, an increase of the tendency of *c *can be due to an increase of the expression rate of gene *c*, or a decrease of the degradation rate of the corresponding protein. In other formalisms these effects are specified by labelling the arcs of the interaction graph with signs (we have used this in the 7th row in Figure 2). A + sign (respectively a - sign) for an interaction of a gene *a *on *b *in the signed interaction graph means informally that the interaction of *a *on *b *is an *activation *(respectively an *inhibition*). However, the exact meaning of the terms activation and inhibition is not clear, especially when several interactions combine on a gene: Does an activation of *b *by *a *forbid an inhibition of *b *by *a *or not? Is an activation of *b *by *a *necessarily observed all along the border associated to the interaction or not? Two properties are used to clarify formally these questions.

The first one, called *additivity*, is the systematic non-strict increase of tendency of *c *when a border is crossed in some predefined direction. In other words the effect on *c *of the interaction composition adds to the effect of all other interaction compositions on *c*. The direction in which the border is crossed for this property is the one given by the passage from a state where the interaction composition is not satisfied to a state where it is satisfied.

The second property, called *observability*, is the existence of a strict increase of the tendency on *c*. This means that the effect on the tendency of *c *exists at least at one crossing point (where the border associated to the interaction composition is crossed in the same direction as the additivity property). In contrast to the additivity property, observability property requires only the existence of an effect somewhere along the border.

To define these effects more formally we introduce for each interaction composition  on *c *a set, denoted by , containing all couples of states (*S*0, *S*1) such that (i) *S*0 is adjacent to *S*1, (ii) *S*0 is a state in the region where  is not satisfied, and (iii) *S*1 is a state in the region where  is satisfied.

**Figure 4:**
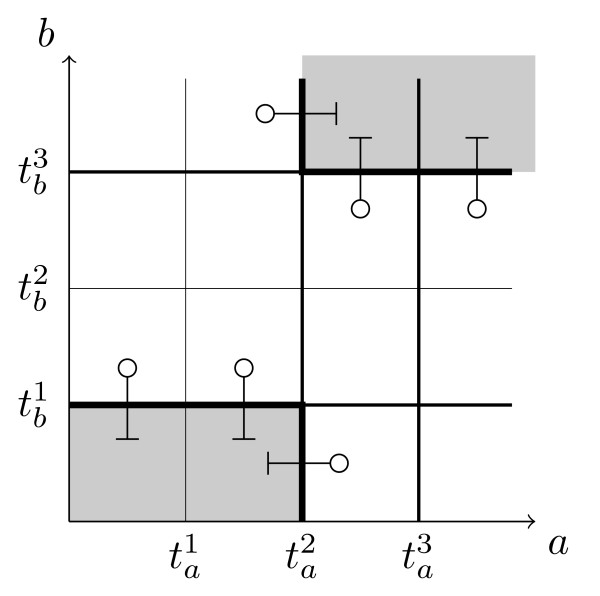
**Graphical representation of  relative to example in Figure 3**.

**Example 4 ***For the interaction composition  of the example given in *Figure 3* we get * = {(⟨0, 1⟩, ⟨0, 0⟩), (⟨1, 1⟩, ⟨1, 0⟩), (⟨2, 0⟩, ⟨1, 0⟩), (⟨1, 3⟩, ⟨2, 3⟩), (⟨2, 2⟩, ⟨2, 3⟩), (⟨3, 2⟩, ⟨3, 3⟩)}. *Each of the couples *(*S*0, *S*1) *of this set is represented in *Figure 4* by a kind of arrow symbol, where the 'o' end is associated to state S*0*, and the *'|' *end to state S*1.

*LG*2 allows to specify that an interaction composition  has an additive effect, denoted by , i.e. that the difference of trend of *c *is positive or zero all along the border defined by . The exact semantics of  is: for every couple (*S*0, *S*1) of  the trend of *c *in *S*0 is less than or equal to the trend of *c *in *S*1. Since the trend of a state is equal to the trend of all the states in the same cellular context, the additivity constraints are expressed as relations between discrete kinetic parameters .

**Example 5 ***For the example in *Figure 2* (with the given order of thresholds) we have * = {(⟨0,0⟩, ⟨1,0⟩), (⟨0,1⟩), ⟨1,1⟩), (⟨0,2⟩, ⟨1,2⟩)}, *and ** due to the negative sign associated to the interaction of y on x*.

*For the first case of interaction compositions on * = {(⟨0,0⟩, ⟨1,0⟩), (⟨1,1⟩, ⟨1,1⟩, (⟨0,2⟩, ⟨1,2⟩)}, , and  = {(⟨0,1⟩, ⟨0,2⟩), (⟨1,1⟩, ⟨1,2⟩)}, .

*For the second case of interaction compositions on y*,  = {(⟨0,2⟩, ⟨1,2⟩), (⟨1,1⟩, ⟨1,2⟩)} *and *. *If this additivity property is true, the only case of activation of y is when x and y are above **and ** respectively*.

*For the third case ** (the same as the first one in the first case because ** is the same) and ** (the same as the first one in the second case). If these aditivity are true, there are two cases of activation of y, one above ** and one above ** and *. *Moreover, the second case of activation is greater than the first one, due to the additivity property *.

If *multi-arcs *are present in the interaction graph (several arcs with the same origin and the same target node) the cellular contexts on each side of the border defined by the interaction composition  are not the same depending on the values of the thresholds associated to the multi-arc. In that case the additivity constraints are relations involving also thresholds. Briefly, the additivity constraint of the interaction composition  is: ∧(*adj*(*l*0, *l*l, *rc*) ⇒  ≥ ) with *adj*(*l*0*, l*1*, rc*) true if it exists a couple (*S*0*, S*1) of  such that  (the cellular contexts *l*0 and *l*1 are non empty, adjacent, and on each side of the border defined by ).

**Example 6 ***For the example in Figure *3*, the additivity constraint of the composition  is:*.

*according to the identifiers l of cellular contexts for b (and so the identifiers of discrete kinetic parameters ). It can be checked with the graphic representation of cellular contexts of b in *Figure 3* that for ** we obtain **. This example shows that specifying additivity properties can be much more compact than working at the level of parameters. Without language LG*2 *we would have to write the above formula*.

In addition to the additivity property, *LG*2 allows to specify that an interaction composition  has an observable effect, denoted by , i.e. that the difference of trend of *c *is strictly positive at least at one position along the border defined by . The exact semantics of  is: for at least one couple (*S*0, *S*1) of  the trend of *c *in *S*0 is strictly less than the trend of *c *in *S*1. To be more explicit, an interaction  can be removed from the interaction graph if neither the interaction composition  (reduced to a single interaction), nor its negation  is observable. Briefly the observability constraint of the interaction composition  is:  with *adj*(*l*0*, l*1*, rc*) true if it exists a couple (*S*0*, S*1) of  such that .

**Example 7 ***For the example in *Figure 3* with ** the constraint ** is *.

### GNBox Features

The core functionality of the GNBox environment is to test, for a given structure of a GRN, the consistency of a set of hypotheses about the behaviours of this GRN (language *LG*1) for several mutant types, about the interaction compositions (language *LG*2), and even directly about the parameters in *P*. GN-Box is able to identify consistent solutions in terms of state variables that define the behaviour (*LG*1) and in terms of parameters of *P*. In cases where the set of hypotheses is inconsistent, it is desirable to determine the possible relaxations of hypotheses to remove the inconsistency. GNBox can identify automatically, among a defined set of questionable hypotheses, all subsets of hypotheses whose relaxation removes the inconsistency (subsets of necessarily false hypotheses). These subsets are represented as disjunctions of conjunctions of negations of hypotheses. For example, the hypotheses *H*1 and *H*2 must be relaxed or the hypothesis *H*3 must be relaxed: (¬*H*1 ∧ *¬H*2) V *¬H*3. Also GNBox automatically identifies, among a defined set of hypotheses, all subsets of hypotheses necessarily true. These subsets are represented by disjunctions of conjunctions of hypotheses. For example, the hypotheses *H*1 and *H*2 are true or the hypothesis *H*3 is true: (*H*1 ∧ *H*2) ∨ *H*3.

## Results and Discussion

### **Application to the immunity control by the **λ **bacteriophage**

The analysis of this network adapted from [7] illustrates mainly the capability of GNBox (i) to express constraints about reachability of states, and (ii) to find the minimal interaction graph consistent with observations.

The λ bacteriophage (or simply λ phage) is a virus that infects the bacterium *Escherichia coli*. The infection starts by the injection of the genetic material of the virus into the cytoplasm of the bacterium. We focus here on two simple observations about the evolution of the bacterium after infection: either the viral DNA is integrated in the genetic material of the bacterium, and the cells continue to divide normally (thus reproducing the phage DNA in the same process), or the genetic material replicates in the cytoplasm of the bacterial cell to create new viral particles and then new viruses until lysis (destruction) of the cell, which leads to the release of new virus particles in the extracellular medium. The first case corresponds to the *lysogenic phase *while the second corresponds to the *lytic phase*. The decision between these two phases is made by a network of viral genes.

**Figure 5:**
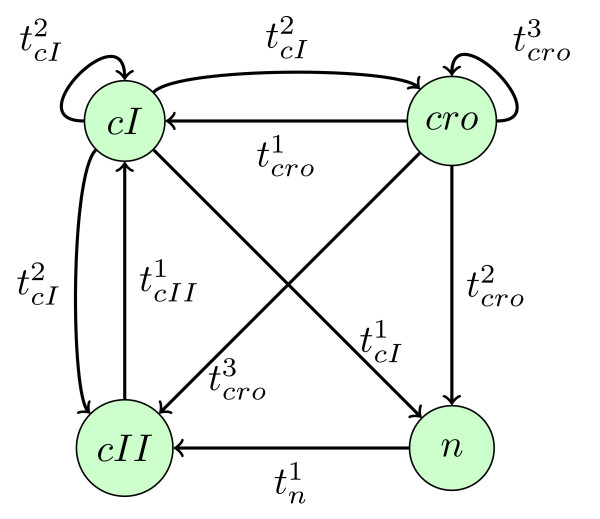
**Interaction graph  for the model about immunity control by the λ phage**.

The model proposed in [7] contains four viral genes denoted by *cI*, *cro*, *cII *and *n*. The gene *cI *is expressed only in the lysogenic phase, *cro *is expressed only in the lytic phase and genes *cII *and *n *are not expressed in both phases. The graph  of interactions between these genes is given in Figure 5. Interactions and interaction compositions (deduced from experimental data) are given in Table 1. We consider the set of all additivity and observability constraints for all these interaction compositions , etc.). In the following we assume that the thresholds  are ordered so That . According to the previous section the set of interactions, the hypotheses about interaction compositions (set of interaction compositions, additivity properties, observability properties) and the hypotheses on threshold values define a parameterized constrained model (couple composed of a focal equation system derived from  and a set of structural constraints). We call it *M*_λ _and it is defined formally by the predicate *model_λ*(*M*_λ_). A state *S *for this model is represented by an ordered list ⟨*S_CI_*, *S_cro_*, *S_cII_*, *S_n_⟩ *of discrete protein concentrations. According to  and hypotheses on threshold values, we have *max_cI _*= 2, *max_cro _*= 3, *max_cII _*= 1, *max_n _*= 1. So the concentration space contains (2+1)*(3+1)*(1+1)*(1+1) = 48 states.

**Table 1:** Interactions and interaction compositions hypotheses for the model about immunity control by the λ phage

species	interactions	interaction compositions
cI		
		
		

cro		
		

cII		
		
		

*n*		
		

The uninfected cell does not have any viral protein and can therefore be represented by the state *S*0 = ⟨*S*0*_cI_, S*0*_cro_, S*0*_cII_, S*0*_n_*⟩ = ⟨0, 0, 0, 0⟩ In the lysogenic phase of the virus-host system the only viral gene expressed is *cI*. This phase is represented by the state *S*1 = ⟨*S*1*_cI_*, *S*1*_cro_*, *S*1*_cII_*, *S*1*_n_*⟩ = ⟨2, 0, 0, 0⟩ such that the concentration of protein *cI *remains equal to its highest value. In the lytic phase the only viral gene expressed is *cro*. In a continuous description this phase is represented by a state which is not contained within a domain, but which is at the border between two adjacent domains. We could introduce in our formalism additional states corresponding to borders between domains. Such states are called *singular states *in [2]. We choose here to stick to the simpler formalism, and we represent the lytic phase as a cycle between the two following states: *S*2 = ⟨0, 2, 0, 0⟩ and *S*3 = ⟨0, 3, 0, 0⟩, such that the concentration of the protein *cro *remains around the highest values 2 and 3 [7]. Biological observations tell us that these two phases must be attractors of the network dynamics, and that they are reachable from the initial conditions. These observations are formalized by Constraint 1 where the lengths of the third and fourth paths for the reachability of the two phases are equal to 48 states, 48 being the total number of states of the state space.

#### Constraint 1

The GNBox environment proves the consistency of this pool of constraints in 2 seconds. All run times mentioned in this article are obtained on a laptop with 2 GB of RAM and running at 2.4 GHz. Moreover GNBox can provide the instantiations of the parameters of *P *that satisfy the pool of constraints.

**Example 8 ***An example of instantiation is:*

**Figure 6:**
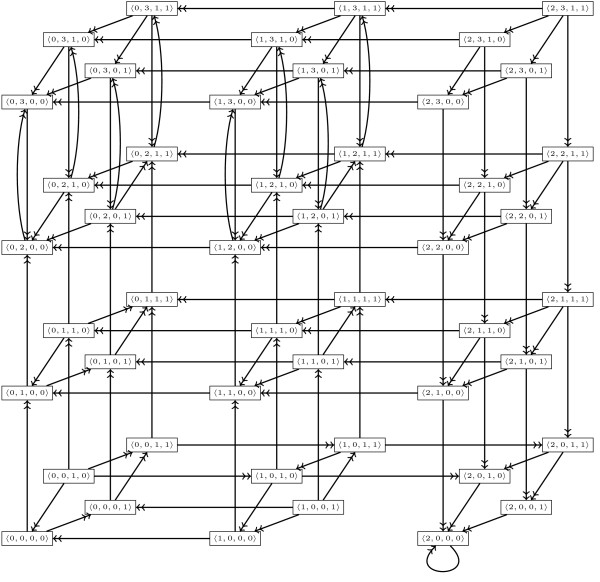
**Set of possible transitions for the instantiation of parameters in Example 8 of the model about immunity control by the λ phage**.

*(remember that the indexes l of discrete kinetic parameters * are set at their creation from the numbering of interactions * See Definition *1*). The set of transitions from S to S', denoted by S ↠ S', for this instantiation is represented in *Figure 6.

An interesting question, akin to reverse-engineering of the network, is: what are the minimal numbers of interaction compositions necessary to get a model consistent with Constraint 1 without specifying any additivity or observability constraints? In other words, we search for the minimal interaction graphs, in terms of interactions, which satisfy the observed behaviors. From a constraint point of view, this problem is specified and implemented in the following way. For each interaction composition on a gene *c*, a Boolean variable is created which means "all pairs of states separated only by this interaction composition have the same evolution tendency for *c*". Then GNBox searches for consistent models such that the number of these Boolean variables which are true is maximized. GNBox finds that only two interactions on *cro *are necessary (in two seconds). So, the minimal interaction graph contains only two interactions on *cro *and no interactions on the other genes. The result is surprising at first sight, but it should be borne in mind that the query contains only poor information about behaviours and no information on the interaction graph (but the limitation to possible interactions), the goal being to infer the minimum graph implied by this information. This does not preclude the existence of other interactions, but means that those are not necessary to account for the behaviours included in the query.

Finally this application lead us to the interesting question of the length *L *of the longest path without cycle in the state space for a given set of hypotheses *Set*. We call this length the *diameter *of the network for *Set*. This knowledge permits to restrict the length of paths in subsequent queries considering a set of hypotheses including *Set*. In our case *Set *is Constraint 1. The diameter for *Set *is 43. This highly combinatorial problem is answered in two queries: one to prove the existence of a solution for a length *L *of 43 in 459 seconds, the other showing inconsistency for a length *L *of 44 in 489 seconds.

### Application to the carbon nutritional stress response in the bacterium *Escherichia coli*

The modeling and analysis of this network is adapted from [8]. It illustrates a case of model revision coming from an inconsistency of the initial set of hypotheses. We performed a similar and more exhaustive study reported in [9]. We show that by an automatic relaxation method over biological constraints we can suggest lines of research to the biologist or, said differently, generate new hypotheses.

Populations of the bacterium *Escherichia coli *grow exponentially in favorable conditions. This state is called the *exponential phase*. In stressing conditions, when food (carbon) starts to be lacking, the populations stop growing and they enter in a state called *stationary phase*, with altered physiology and morphology. The phenomenon is reversible: the population can return to the exponential phase if the conditions become favorable again.

**Figure 7:**
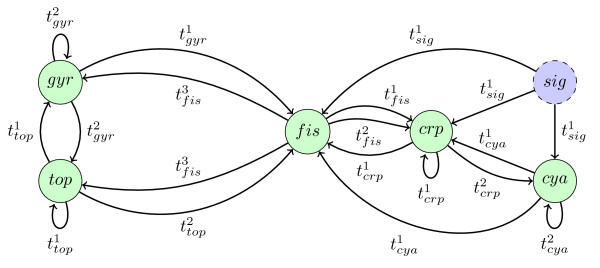
**Interaction graph  for the model about carbon nutritional stress in *E. coli***.

The model, proposed in [8] and adapted to our formalism, contains one input node *sig *(signal, 0 in the absence of stress and 1 in the presence of stress) and five species: *crp*, *cya*, *fis*, *gyr *and *top*. The interaction graph  is given in Figure 7 where the input *sig *is represented by a dotted circle filled in blue. Interactions and interaction compositions are given in Table 2. Moreover we consider the set of all additivity and observability constraints for all interaction compositions. As before, the thresholds  are ordered and equal to *p*. The model obtained from all these hypotheses is denoted by *M_coli_*. Thus we have *max_crp _*= 2, *max_cya _*= 2, *max_fis _*= 3, *max_gyr _*= 2 and *max_top _*= 2 (for the input *sig *we have *max_sig _*= 1). We obtain a concentration space of (2 + 1) * (2 + 1) * (3 + 1) * (2 + 1) * (2 + 1) = 324 states.

**Table 2:** Interactions and interaction compositions hypotheses for the model about carbon nutritional stress in *E. coli*

species	interactions	interaction compositions
*crp*		
		
		
		
		

*cya*		
		
		

*fis*		
		
		
		
		

*gyr*		
		
		

*top*		
		
		

The exponential phase and the stationary phase are modeled by two states, respectively *S^ns ^*(*ns *for "not stressed") and *S^s ^*(*s *for "stressed"). As stated in [8], there exists partial knowledge about these states:

#### Constraint 2

Note that only three components are instantiated in each state and that there is a relationship between the two others which expresses the fact that the super-coiling of DNA is higher in the exponential phase. To model the presence or the absence of stress we use two models: a model  without stress (*sig *= 0) and a model  with stress (*sig *= 1). These two models are the same biological model *M*_*coli *_in different conditions. So they share the same discrete kinetic parameters . In absence of stress the system beginning in the stressed state *S^s ^*can reach the non-stressed state *S^ns^*, which is steady. In presence of stress the system beginning in *S^ns ^*can reach *S^s^*, which is steady. We formalize that by:

#### Constraint 3

where *L *is the length of the third and fourth paths for the reachability of the two steady states. In the following queries we choose *L *= 10 and *L *= 100 to compare performance, but if we want a general query without any limitation on *L *we should choose *L *= 324 (the total number of states of the model) but the amount of memory needed to generate the pool of constraints becomes very large. We point out here that for such queries involving paths, this approach is limited to networks of medium size.

With GNBox we prove that the pool of constraints composed of constraints for models  and  Constraint 2 and Constraint 3 is inconsistent in 2 seconds for path length *L *= 10 states, and 13 seconds for path length *L *= 100. In fact just imposing the existence of two steady states gives an inconsistency in less than 1 second, thus proving that the constraint pool is inconsistent whatever the value of *L*. In [8] it is noted that the proposed instantiated model is indeed inconsistent. Here we prove in addition that there exists no other instantiation of the discrete kinetic parameters (accepting the interaction compositions hypotheses) able to restore consistency. In other words it is proved that this network architecture with these hypotheses on interaction compositions is incompatible with the observations. It is thus necessary to revise the model. In [8] the authors suggest that a regulator or an interaction may be missing in the model. Here, instead, we keep the interaction graph  as it is, and try to change the way interactions are composed. The set of unreliable hypotheses is the set of all additivity and observability properties about interaction compositions. We allow the relaxation of these hypotheses and we obtain the property  in 7 seconds with a path length *L *= 10, and in 830 seconds with a path length *L *= 100. Discussions with the biologist lead to the conclusion that it is not acceptable to relax the additivity property of the first composition on *gyr*, . This suggests that the composition on *top*, , is the one which is not additive and consequently that it is possible to observe an inhibition of *top *by *fis*. This inhibition effect is actually observed for another kind of stress. In [10] it is said: "when Fis levels are low, hydrogen peroxide treatment results in topA activation". This means that *fis *acts in some cellular contexts as an inhibitor of *top*. This paper shows that the protein *fis *can indeed play an inhibitory role on *top *in some contexts, and it thus gives support to the new hypothesis that *fis *plays an inhibitory role in the response to nutritional stress. It is remarkable that this pool of constraints is inconsistent given that the number of adjustable parameters is relatively high. We insist here on the fact that inspection of the constraint pool did not allow to resolve manually this inconsistency.

Finally, it appears that the hypotheses of interaction compositions on *top *are not well supported by experiments, and we propose to determine the necessarily observable compositions of the type  and (). The rationale for limiting the compositions to basic types (signed interactions) is to provide easily interpretable results in terms of the interaction graph  complemented with interaction signs (corresponding to the choice between activation and inhibition). This allows to determine for example whether there are unnecessary arcs in . On the other hand this restriction still allows to guide the user in the choice of hypotheses about interaction compositions. We conserve all the previous hypotheses except the ones about the interaction compositions on *top*. We consider a new set of these six interaction compositions for *top*: . Finally we challenge the observability constraints onto them to find which of them are necessary for these hypotheses. GNBox returns the property  (observability property of  or observability property of) in 4 seconds with a length of path *L *= 10, and the same formula in 60 seconds with a length of path *L *= 100. This indicates that any solution of all constraints (except the hypotheses of composition on *top*) has the property  or the property . This result provides an essential information to help the biologist to make additional hypotheses about interaction compositions.

### Application to the gap-gene module of the segmentation of the *Drosophila melanogaster *embryo

In the first hours after fertilization, the embryo of the fly *Drosophila melanogaster *undergoes segmentation along the anteroposterior axis (head to tail). The embryo is partitioned into segments, each segment being made of cells characterized by specific levels of a set of proteins. Segmentation takes place in several successive stages controlled by distinct genetic modules. Here we focus on the gap-gene regulatory module.

The modeling and analysis of this network illustrates the expression of steady states in several segments of the embryo for the wild type and several mutant types, and the search for the minimum number of thresholds necessary to account for all the observations. The initial model is adapted from [11,12]. Although this model is not the most recent available, it is convenient for our purpose. The resolution of this query provides a set of minimal models (in terms of number of thresholds) consistent with a set of very diverse observations. The connection between the observations for all these models (one for each mutant type and for each segment) adds a new level of complexity.

**Figure 8:**
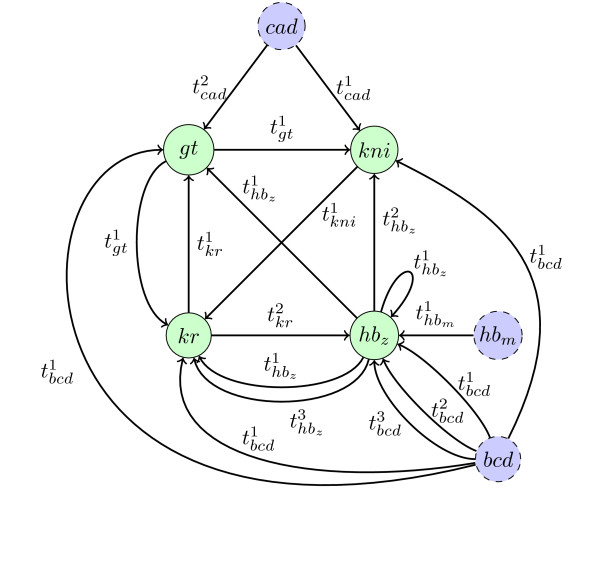
**Interaction graph  for the model about gap-gene module of the segmentation of the *D. melanogaster *embryo**.

The model, proposed in [11,12], controlling the gap-gene module contains seven genes: Giant denoted by *gt*, Hunchback zygotic denoted by *hb_z_*, Hunchback maternal denoted by *hb_m_*, Krüppel denoted by *kr*, Knirps denoted by *kni*, Bicoid denoted by *bcd*, and Caudal denoted by *cad*. The genes *bcd*, *hb_m _*and *cad *are input genes: they influence other genes but are not influenced by any gene. Stocks of maternal mRNA and proteins are accumulated at specific places of the egg before fertilization. These molecules generate gradients along the anteroposterior axis. In the model these quantities are represented by input parameters (one for each chemical species and each region). The interaction graph  between these genes is given in Figure 8 where input genes are represented by dotted circles filled in blue. Interactions and interaction compositions are given in Table 3. Moreover we consider the set of all additivity and observability constraints for all interaction compositions. The modeling in [11,12] takes into account four adjacent segments along the anteroposterior axis, denoted by *A*, *B*, *C *and *D*. Genetic experiments produced information on the concentration of the gap-gene proteins for the wild type, denoted by *wt*, and nine mutants. The mutants are: knock-out (KO) on *gt *(the focal value of the *gt *component is 0 everywhere) denoted by *gt*0, KO on both *hb_z _*and *hb_m _*denoted by *hb*0, KO on *kr *denoted by *kr*0, KO on *kni *denoted by *kni*0, KO on *bcd *denoted by *bcd*0, KO on *hb_m _*denoted by *hbm*0, KO on *cad *denoted by *cad*0, ectopic expression equal to 1 on *gt *(the focal value of the *gt *component is everywhere equal to 1) denoted by *gt*1, ectopic expression equal to 1 on *kni *denoted by *kni*1. We define a model *M^R,T ^*for each segment *R∈ *{*A, B, C, D*} and each type *T ∈ *{*wt, gt*0*, hb*0*, kr*0*, kni*0*, bcd*0*, cad*0*, hbm*0*, gt*1*, kni*}. For example, *M^B^,^gt0 ^*corresponds to the model of the mutant type *gt*0 in segment *B*. The input parameters, discrete kinetic parameters and threshold parameters, between models are linked by introducing equality constraints between them, as explained in the section on the formalization of Thomas networks. Thus it would be redundant to impose constraints about interaction compositions for mutant types (the corresponding constraints for the wild type are sufficient). Obviously, these constraints lead to exactly the same threshold and discrete kinetic parameters for the four models associated to the four segments and each mutant.

**Table 3:** Interactions and interaction compositions hypotheses for the model about gap-gene module of the segmentation of the *D. melanogaster *embryo

species	Interactions	interaction compositions
*gt*		
		
		
		

*hb_z_*		
		
		
		
		
		

*kr*		
		
		
		
		

*kni*		
		
		
		

The concentrations of the proteins produced by input genes *bcd*, *hb_m _*and *cad *for each region *R *and each mutant type *T *are respectively denoted by  and . We impose in Constraint 4 that the inputs in the mutant types are equal to those of the wild type, except in the cases where some input genes themselves are mutated. This exception is due to the fact that the inputs come from the mother system, so only a mutation in this system can change the concentration value of the corresponding input.

#### Constraint 4

Moreover, we have inequality constraints between the thresholds for this model:

#### Constraint 5

The observations relate to the existence of one steady state by mutant type and by segment with some properties between these states. The steady states are represented by ordered lists of four protein concentrations:  for each region *R *and each mutant type *T*. The constraint associated to the observation of the steady state of each region *R *and each type *T *is:

#### Constraint 6

The gradients of maternal origin mentioned above are used by the cell to derive positional information. To represent these gradients, the antero-posterior axis is partitioned into segments, each segment being identified by a combination of values of the input molecules *bcd*, *hb_m _*and *cad*. We impose that the combinations of input quantities are different for all pairs of segments for the wild type:

#### Constraint 7

Table 4 shows the constraints between the concentrations of the steady states in regions *A*, *B*, *C *and *D *for each species and for each mutant; the bold font indicates a change of constraint compared to the wild type. All these constraints come from the interpretation of data in [12]. The first column of the table gives the mutant type, the second the genes, the third, fourth, fifth and sixth represent the steady state of the region *A*, *B*, *C *and *D*, respectively. Inequality symbols appear between the columns labeled A, B, C, D. They indicate constraints between concentrations of steady states of adjacent segments. Finally the two last columns give other constraints involving concentrations in segments that are not necessarily adjacent and comments about the differences compared to the wild type.

**Table 4:** Constraints between stationary states and between input parameters for each region and each mutant type for the model about gap-gene module of the segmentation of the *D. melanogaster *embryo

Type	species	A		B		C		D	**supp. constr**.	Comments
*wt*	*bcd*		≥		≥		≥			
	*hb_m_*		≥		≥		≥			
	*cad*		≤		≤		≤			
			
	*gt*		>				<			
	*hb_z_*		≥		≥		≥			
	*kr*		<		≥		≥			
	*kni*		≤		<		>			

*gt*0	*gt*		≥		≥		≥			Knock-out
	*hb_z_*		<		≥		≥			
	*Kr*		≤		<		≤			*Kni *expands into *D*

*hb*0	*bcd*		≥		≥		≥			Knock-out
	*hb_m_*									
	*cad*		≤		≤		≤			
			
	*gt*									*gt *expand into *BC*
	*hb_z_*									Knock-out
	*kr*									loss of *kr *into *BC*
	*kni*									loss of *kni *into *BC*

*kr*0	*gt*									*gt *expands into *BC*
	*hb_z_*		≥		≥		≥			
	*kr*									Knock-out
	*kni*									loss of *kni *into *BC*

*kni*0	*gt*						<			
	*hb_z_*		>		≥		≥			
	*kr*		≥		≥		≥			
	*Kni*		<							increase of *kr *into *c *knoct-out

*bcd*0	*bcd*									Knock-out
	*hb_m_*		≥		≥		≥			
	*cad*		≤		≤		≤			
			
	*gt*						<			loss of *gt *into *A*
	*hb_z_*									loss of *hb*_*z *_into *ABC*
	*kr*									loss of *kr *into *BC*
	*kni*		≥		≥		>			*kni *expands into *AB*

*hbm0*	*bcd*		≥		≥		≥			
	*hb_m_*									Knock-out
	*cad*		≤		≤		≤			
			
	*gt*		>				<			
	*hb_z_*		≥		≥		≥			
	*kr*		<		≥		≥			
	*kni*		≥		<		>			

*cad0*	*bcd*		≥		≥		≥			
	*hb_m_*		≥		≥		≥			
	*cad*									
			
	*gt*		>							knock-out lass of *gt *into *D*
	*hb_z_*		≥		≥		≥			
	*kr*		<		≥		≥			increase of *kr *of into *C*
	*kni*									lass of *kni *into *C*

*gt*1	*gt*									ectopic expression
	*hb_z_*		≥		≥		≥			
	*kr*		<		≥		≥			
	*kni*									lass of *kni *into *C*

*Kni*1	*gt*		≥		>		<			activation of *gt *into *B*
	*hb_z_*		≥		≥		≥			
	*Kr*						≥			
	*kni*									lass of *kr *into *B* ectopic expression

We note in the following *C_gap _*the set of constraints associated to the existence of the steady states of the 4 regions for each of the 10 types (composed of constraints defining and linking the models *M^R,T^*, Constraint 4, Constraint 5, Constraint 6, Constraint 7, and constraints in Table 4).

If we add to *C_gap _*the constraint represented in Table 5 about the instantiation of steady states for all types, input parameters for the wild type according to the second table of [12], and the constraint ∧_*c *_∧_*p *_ we obtain a consistency in 11 seconds.

**Table 5:** Constraints of instantiation of stationary states according to the second table in [12] for each region and each mutant type for the model about gap-gene module of the segmentation of the *D. melanogaster *embryo

type	species	A	B	C	D
*wt*	*bcd*	3	2	1	0
	*hb_m_*	1	1	0	0
	*cad*	0	0	1	2
	
	*gt*	1	0	0	1
	*hb_z_*	3	2	1	0
	*kr*	0	2	1	0
	*kni*	0	0	1	0

*gt*0	*gt*	0	0	0	0
	*hb_z_*	3	2	1	0
	*kr*	0	2	1	0
	*kni*	0	0	1	1

*hb*0	*gt*	1	1	1	1
	*hb_z_*	0	0	0	0
	*kr*	0	0	0	0
	*kni*	0	0	0	0

*kr*0	*gt*	1	1	1	1
	*hb*_*z*_	3	2	1	0
	*Kr*	0	0	0	0
	*Kni*	0	0	0	0

*kni*0	*gt*	1	0	0	1
	*hb_z_*	3	2	1	0
	*kr*	0	2	2	0
	*kni*	0	0	0	0

*bcd*0	*gt*	0	0	0	1
	*hb_z_*	0	0	0	0
	*kr*	0	0	0	0
	*kni*	1	1	1	0

*hbm*0	*gt*	1	0	0	1
	*hb_z_*	3	2	1	0
	*kr*	0	2	1	0
	*kni*	0	0	1	0

*cad*0	*gt*	1	0	0	0
	*hb_z_*	3	2	1	0
	*kr*	0	2	2	0
	*kni*	0	0	0	0

*gt*1	*gt*	1	1	1	1
	*hb_z_*	3	2	1	0
	*kr*	0	1	1	0
	*kni*	1	1	1	1

*kni*1	*gt*	1	1	0	1
	*hb_z_*	3	2	1	0
	*kr*	0	0	1	0
	*kni*	1	1	1	1

It appears in the second figure of [11] that there is no auto-interaction onto *hb_z_*. In fact, after discussion with D. Thieffry, a synergy between Hunchback and Bicoid on the activation of Hunchback has been reported, and *hb_m _*and *hb_z _*are the same species in distinct compartments. This explain the interaction compositions onto *hb_z _*with indexes 3, 4 and 5. If we do not consider this auto-interaction in *C_gap _*by replacing the last three interaction compositions onto *hb_z _*by  and , and we still consider the constraint in Table 5 and the constraint ∧_*c *_∧_*p *_, we obtain an inconsistency in 7 seconds.

So we consider in the following the proposed model with this auto-interaction onto *hb_z _*(in order to have a similar model to those in [11,12]). Obviously, *C_gap _*alone (without the constraint represented in Table 5 and the constraint ∧_*c *_∧_*p *_) is consistent according to the first query.

In previous applications the thresholds  are instantiated and equal to *p*, *p *taking a value between 1 and *max_c_*. This implies that the concentrations of *c *can take values between 0 and *max_c_*. Insofar as the subdivision of the concentration space is only speculative, it is interesting to ask what is the smallest number of distinct thresholds necessary to get a model consistent with the observations. The extreme case would be the satisfaction of all observations and hypotheses with one threshold per component, i.e. with a Boolean model. It appears that *C_gap _*plus ∧_*c *_∧_*p *_ is inconsistent in 2 seconds. We can check easily this inconsistency: the constraint  is inconsistent with the observability constraint of  because no state *S *satisfies  i.e. the condition .

To identify the minimum number of different thresholds needed to satisfy all the observations and hypotheses, we must build a query using the method of relaxation of constraints. But in this case, the relaxation takes place on the number of thresholds in 1*..max_c _*for each component *c*.

To summarize, we challenge the hypotheses about the number of thresholds for all components. From a constraint point of view, this problem is specified and implemented in the following way. We introduce Boolean variables *B_j,c _*equivalent to "the number of thresholds for *c *is less or equal to *j*", *j *being an integer in the interval 1..*max_c _*-1. So we get six Boolean variables in our case:  and  for *hb_z_*, *B*_1, *kr *_for *kr*, *B*_1, *bcd *_and *B*_2, *bcd *_for *bcd*, *B*_1, *cad *_for *cad*. Then GNBox searches for models consistent with the set of constraints defined above such that the number of these Boolean variables which are true is maximum. GNBox finds in 22 seconds that the only Boolean variables to be false are  and *B*_2, *cad*_. This indicates that *hb*_*z *_must have at least 2 different values of thresholds, and *cad *must have at least 2 different values of thresholds.

The last query gives, in 17 seconds, two possible instantiations of the  accepting *C_gap _*and the minimal number of thresholds given by the previous query: . One remarks that the three thresholds of *hb_z _*share only two values.

## Conclusions

Our methodology is composed of two parts: (i) a declarative constraint-based approach; (ii) a formalism for the description of the dynamics of discrete networks. We have presented here applications involving gene regulatory networks whose behaviour is satisfactorily represented in the formalism of R. Thomas. But it is important to note that the methodology can be applied to many other types of dynamical rules, such as Hopfield-like networks, Boolean networks with parallel, sequential or block-sequential updating. The only requirement is that the dynamical rules should be expressed as constraints on finite-domain variables. The potential domain of application of this methodology is thus much larger than just gene regulatory networks.

The Thomas' networks have largely been applied to the analysis of GRNs, for example those described in [7,13-15] or those described in [8,16,17] which use a very similar qualitative formalism.

Several modeling and simulation tools of biological regulatory networks (for example GINsim [18], BIOCHAM [19], GNA [20]) are used in combination with model checkers (NuSMV, CADP) and based on diverse formalisms (logic, Petri nets, ODEs). The idea is to add to the simulation functionality a formal verification functionality to check, or optimize [21], the fitness between the simulated and the observed behaviours.

Three types of abstractions are available in BIOCHAM, among which ordinary differential equations and Boolean networks. The inference of parameters is based on the technique of model-checking and the definition of a continuous degree of satisfaction of a temporal logic formula formalizing some observation on behaviour. This permits to find biochemical kinetic parameter values which are optimal with respect to a set of biological properties. Moreover it is possible to find the effect of parameter variations on the robustness of a behaviour specification [22]. Our work differs significantly in that it focuses to face the problem of incomplete knowledge to produce, by a constraint-based process, a class of models from which it is expected to design new experiments.

A steady state search module, including the so-called singular states, exists in GNA based on an integration of the SAT solver SAT4J [23]. This integration of a constraint approach avoids the generation of all the transitions to identify the steady states. But in contrast to our work aiming at providing general queries, [23] focus on the search of steady states, and only in the case of completely instantiated models (kinetic parameters instantiated and order of the thresholds predefined). The work in [24] search the same steady states with a CSP (Constraint Satisfaction Problem) formalization, the performances are worse than in [23]. In our work, we can write easily queries to identify steady states, and even cycles of length smaller than some predefined value. In addition in our case the kinetic parameters and the orders between thresholds can be only partially known. As explained here, other much more sophisticated queries are available, although in the current version we do not include singular states.

The formal approach proposed here modifies deeply the way to proceed in the building and in the exploration of genetic and biochemical networks, first by avoiding the usual trial-and-error procedure, and second by putting the emphasis on sets of solutions, rather than a single consistent solution arbitrarily chosen in a set. Last, the constraint approach lends to a unified description of network architecture and network behaviour, as both are described in terms on formal constraints. The knowledge available to initiate the modeling of a given phenomenon is generally sparse with respect to the complexity of the behaviour of the underlying networks. It is thus essential to exploit consistently, efficiently, and in a joint manner, every bit of experimental information. The representation of knowledge in terms of constraints is a way to achieve, at least to some extent, this goal.

Our environment GNBox implements a wide panel of functionalities: simulations, consistency proof, relaxation in case of inconsistency, search for a minimal model, prediction of properties in case of consistency. This last functionality generates properties which are verified by all solutions of the constraint pool. In line with what we said above, note that such properties are really supported by data. This contrasts with the usual practice of using just one solution to make prediction, neglecting the existence of other solutions. Properties of the selected single model should not be considered as true predictions.

We have presented three biological applications illustrating the use of most of these functionalities. These applications involve networks containing about 5 species and 15 possible interactions, and with set of hypotheses and observations without systematic instantiation of threshold parameters, with a large range of types of behaviours. In the third application the queries involve several structurally related models in order to incorporate knowledge about wild-type and mutant behaviour, in four segments of the embryo. The set of constraints generates a dense network of dependencies between the variables. The performances of GNBox are good for the different types of queries presented in the three applications. The most computer-intensive queries are those involving paths. For such queries our approach is limited to networks of medium size.

The perspectives are governed by the biological problems. The methodologies and technologies employed must be chosen according to these problems. A first perspective is to prioritize biological experiments. For example, consider a situation in which the state of knowledge is such that the number of consistent instantiated models is still large, and it is possible to perform double knock-out experiments. In such situation it would be interesting to be able to determine the most informative choice of pairs of genes to target for knock-out, an informative experiment being one which will potentially add non redundant constraints and thus reduce the set of solutions. Another perspective is to refine the abstraction of the discrete behaviours: for example by taking into account the trajectories sliding along the thresholds [17,25], and taking into account the difference of delays of chemical reactions [26-28]. Another perspective is the extended repairing consistency techniques adding species, related to the problem of composing networks [29].

## Authors' contributions

The methods and applications was mainly developed by FC on theoretical foundation and ideas provided by EF and LT. All authors equally wrote this manuscript and approved it.

## Supplementary Material

Additional file 1 **GNBox**. Click here for file
